# A Quadruped Robot with Three-Dimensional Flexible Legs

**DOI:** 10.3390/s21144907

**Published:** 2021-07-19

**Authors:** Wenkai Huang, Junlong Xiao, Feilong Zeng, Puwei Lu, Guojian Lin, Wei Hu, Xuyu Lin, Yu Wu

**Affiliations:** 1 School of Mechanical & Electrical Engineering, Guangzhou University, Guangzhou 510006, China; smallkat@gzhu.edu.cn (W.H.); 1707200071@e.gzhu.edu.cn (J.X.); 1807700032@e.gzhu.edu.cn (F.Z.); 2111907042@e.gzhu.edu.cn (P.L.); 2112007108@e.gzhu.edu.cn (G.L.); 1707700083@e.gzhu.edu.cn (W.H.); 1807700012@e.gzhu.edu.cn (X.L.); 2 Laboratory Center, Guangzhou University, Guangzhou 510006, China

**Keywords:** quadruped mobile robots, rigid–flexible coupling, motion planning and control

## Abstract

As an important part of the quadruped robot, the leg determines its performance. Flexible legs or flexible joints aid in the buffering and adaptability of robots. At present, most flexible quadruped robots only have two-dimensional flexibility or use complex parallel structures to achieve three-dimensional flexibility. This research will propose a new type of three-dimensional flexible structure. This passive compliant three-dimensional flexibility reduces the weight and complex structure of the robot. The anti-impact performance of the robot is verified by a side impact experiment. The simulation and experiments show that the robot still has good stability even under a simple algorithm and that the flexible leg can reduce the impact on the quadruped robot and improve the environmental adaptability of the robot.

## 1. Introduction

At present, human living areas are relatively concentrated. Many wild areas in the world are still mysterious and dangerous, with particularly complex terrain. In most places, even human beings cannot cope with the complex environment, which can easily cause risks, as human beings can easily be attacked by wild animals or be trapped and fall in the wild. At this time, it is important for robots to replace human beings in field explorations or extreme environments to perform tasks that are otherwise difficult, thus aiding in safer field information obtainment. Additionally, with the application of robots more and more widely, robots will face unknown and complex environments, so physical interaction with the environment is inevitable [1]. In the existing field of mobile robots, the fault tolerance of multi-legged robots in complex environments is higher than that of wheeled and tracked mobile robots [2], which makes them highly valued.

Nowadays, the development of multi-legged robots is rapid and includes the bipedal humanoid [3,4,5], the hexapod spider [6,7], and the quadruped robots, such as bionic cheetah, bionic dog, among others. Among these, the quadruped robot is considered to be the best in terms of stability and control difficulty [8]. Compared to the traditional rigid quadruped robot, the quadruped robot with flexible legs or joints is the current development trend. Specifically, the damper of the robot plays an important role in reducing the vibrations of the robot and increasing the stability of the robot. In fact, in real life, some quadrupeds have fat, muscle, and meat mats on their legs to improve their adaptability to the complex environment of the wild. The research on the quadruped robot is mostly based on the principle of bionics, which uses a variety of dampers to improve the body of the robot, but different dampers have varying advantages and disadvantages. Yang [9] researched the hydraulic-driven quadruped robot. Semini et al. [10] developed the HYQ hydraulic quadruped robot and the newly developed hyq2max [11]. Although the hydraulic drive has a strong bearing capacity, it will increase the weight of the robot, and the leakage can be very dangerous [12], resulting in low efficiency and high costs. Some researchers have also studied the pneumatic quadruped robot. Wait and Goldfarb proposed a quadruped robot with pneumatic cylinders instead of artificial muscles [13]. Moreover, the cheetah-like high-speed robot [14,15] is safer and faster than a robot driven by hydraulic pressure. However, if only the cylinder is used as the tendon, the flexibility of the robot is only in the two-dimensional plane. If multiple cylinders drive a leg in parallel, it will not only increase the weight of the robot but also increase the complexity of the drive. Another design method is to use the spring to achieve joint flexibility, which provides shock absorption and reduces the bottom impact through the passive compliance of the spring [16,17]. Additionally, the spring and connecting rod can be used to make a flexible leg directly [18,19,20]. The spring is used as a joint damper [21]. Nizami et al. [22] used a spring with a scissor mechanism to achieve flexible legs. However, the spring has the following disadvantages: natural vibrations, the easy transmittance of low-frequency vibrations, and a long attenuation time during the buffering process. Compared with using the spring’s passive adaptability, the flexible leg that uses an actuator to control the leg’s telescopic stiffness [23] has a stronger active adaptability, but its controller is also very complex. Moreover, the spring damper will produce vibration problems, which are difficult to eliminate due to the inherent properties of the spring.

Regarding the adaptability of quadruped robots working in a field environment, only through the two-dimensional plane flexibility is not sufficient. Although the control algorithm can be used to solve the stability problem when the robot is subjected to external forces from other directions, it is often more complex. Parallel structures can also be used to achieve three-dimensional flexibility, such as parallel spring legs [24], but the spring will still have the disadvantages mentioned earlier. Another method is to use a parallel cylinder or hydraulic cylinder to drive the quadruped robot toward active compliance, but this will greatly increase the complexity of the control and the weight of the robot’s body, so that the robot cannot be small and medium-sized. Therefore, this paper proposes a new structure using three-dimensional passive compliance to solve this problem. The structure has strong adaptability, is light weight, and features a convenient adjustment of the stiffness and leg length. Compared with other rigid–flexible coupled quadruped robots, the robot can also have good stability in all directions without a complex algorithm. At present, many challenges faced by medium-sized quadruped robots are related to these characteristics in regard to the above, i.e., light weight, simple to control, and good stability.

The main contributions of this paper are as follows:

- A new three-dimensional flexible leg is designed and its material is characterized;
- The dynamics and gait planning of the robot are designed, and the effectiveness of the technology is verified via simulation;
- The experiments show that the robot has the characteristics of stability and fast recovery to a stable state when it is impacted by the side.

The remainder of the paper is organized as follows: Section 2.1 and Section 2.2 present the structure and characterization of flexible legs. The kinematic and dynamic models of the robot are presented in Section 2.3. Section 2.4 presents gait planning. The hardware part of the robot is explained in Section 2.5. Then, the results are shown in Section 3. We discuss the results in Section 4. Finally, the conclusions are drawn in Section 5.

## 2. Materials and Methods

### 2.1. Design Principle of a Rigid–Flexible Coupling Leg

**Figure 1:**
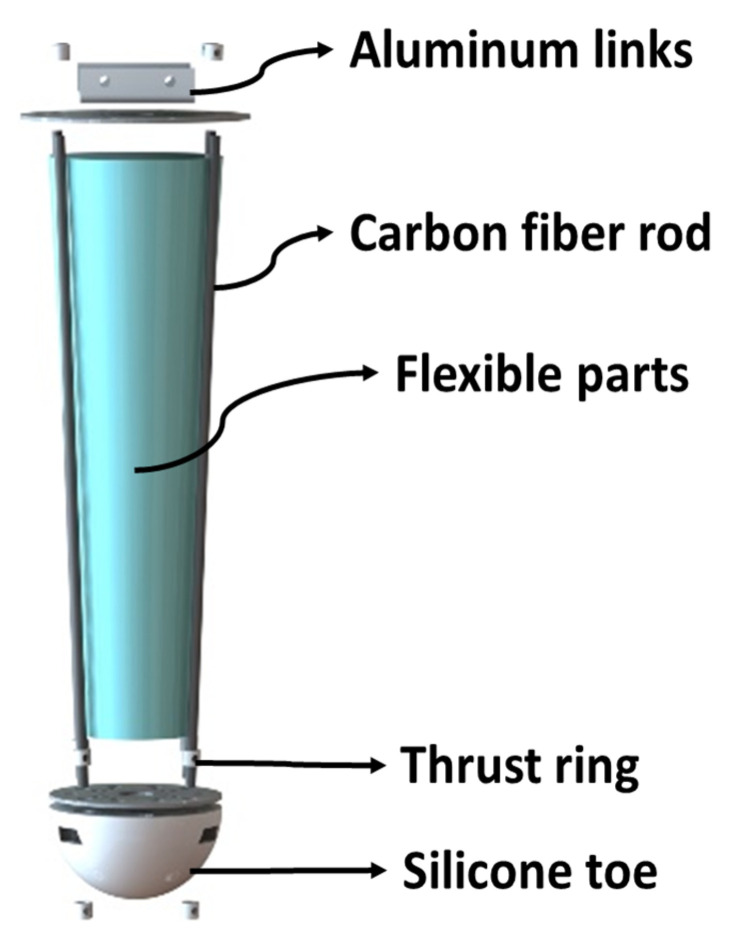
Explosion view of rigid and flexible coupling legs.

The structure of the leg is shown in Figure 1. The motor connector is made of aluminum plate, thrust rings are used to limit the stiffness range of the flexible part, and the carbon fiber rods are used to limit the lateral deformation of the flexible part. The flexible part is composed of a plurality of air bags, a carbon fiber board is used to support the flexible air bag, and flexible silicone toes provide flexible contact between the end of the robot’s leg and the spherical surface of the ground. At present, most quadruped robots use silicone toes to make spherical contact between the end of the foot and the ground, and toes will be greatly impacted when the end of the foot makes contact with the ground during the movement of the robot. Using a flexible ball as the sole is similar to the meat ball of an animal’s sole, which is equivalent to an additional passive degree of freedom, i.e., it can reduce the impact.

The design principle is to make the legs fit with flexible air bags and rigid bones. Purely flexible animals are adaptable but move very slowly in reality. Although the purely rigid structure is very fast, in order to improve the stability of the robot, we can only carry several sensors and use complex control algorithms. The rigid–flexible coupling structure takes into account the adaptability of flexibility and the speed of rigidity. In this design, the low density and high compression characteristics of the air bags can be used to support the body and reduce the weight of the whole machine. Moreover, each air bag can be compressed independently. The carbon fiber rods can not only limit the flexible part of the legs but also provide some stiffness and cooperate with the thrust rings. Thus, this structure can adjust the stiffness and leg length by adjusting the position of the thrust rings. When there are thrust rings on both sides of a carbon fiber board, the lateral deformation of flexible legs can be limited. First, a quadruped robot model with only lower leg flexibility is discussed. The flexible leg experiment can be seen in video (https://www.bilibili.com/video/BV1QK4y1u7iw/ (accessed on 7 July 2021)) provided in the Supplementary Materials of this research.

### 2.2. Characterization of the Flexible Leg

**Figure 2:**
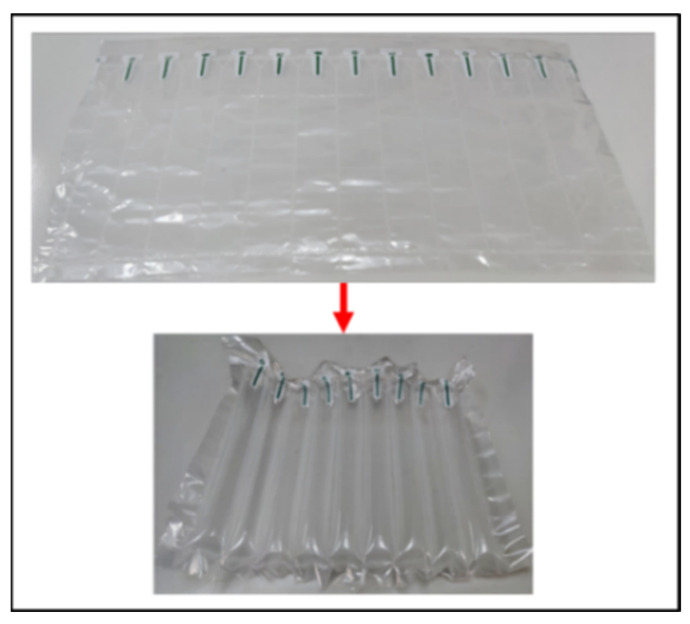
Flexible leg material.

The flexible leg is made of 150 mm-long shockproof air bags made by Zhejiang Enron Packaging Materials Co., Ltd. This material is made of nine layers of coextrusion original film PE (polypropylene) + PA (nylon). It features a design with strong compression and air leakage prevention, making it easy to use, and it can be used just by cutting and filling. The company has certified the material by Societe Generale de Surveillance S.A (SGS). It can bear the weight of 80 kg and will not leak for several months. The method of use is shown in Figure 2. Shockproof air bags are usually used for the filling and packaging of express delivery, and they are not only low cost but also recyclable.

**Figure 3:**
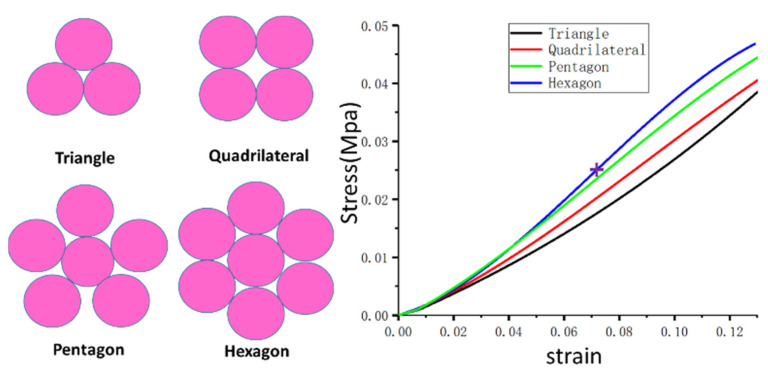
Stress–strain curves of different winding methods.

In this paper, the stress and strain of a flexible leg with different winding methods are tested. The results of the compression experiment are shown in Figure 3. It can be seen that the different winding methods affect the elastic modulus characteristics. Increasing the number of bundles will increase the elastic modulus and hinder its extrusion. When the whole flexible part is surrounded by carbon fiber rods, the gap in the flexible air column is smaller. The elastic modulus of pentagons and hexagons is higher than that of triangles and quadrangles, and the former has more uniform compressive strength in all directions. Due to the low elastic modulus of the front half of the air column, the legs will be too soft, which will lead to greater robot walking errors. Therefore, in the actual assembly, the air column will be preloaded, and the initial compression of the air column can be changed by adjusting the position of the thrust rings so as to adjust the stiffness of the flexible leg. In this study, based on the leg size and elastic modulus requirements, the hexagonal mode is selected for winding. Moreover, the length is limited to 139 mm, as shown with the purple cross symbol in Figure 3, because the deformation of the flexible leg caused by the robot during the process of motion after loading the preload is not very large. In order to facilitate a dynamic calculation and simulate a solution, the elastic modulus of the flexible leg can be regarded as a constant E = 0.35 Mpa, through the stress–strain experiment Poisson’s ratio is v = 0.1, and through the immersion method experiment the density can be calculated as ρ≈10 kg/m^3.

### 2.3. Kinematics and Dynamics of the Proposed Robot

**Figure 4:**
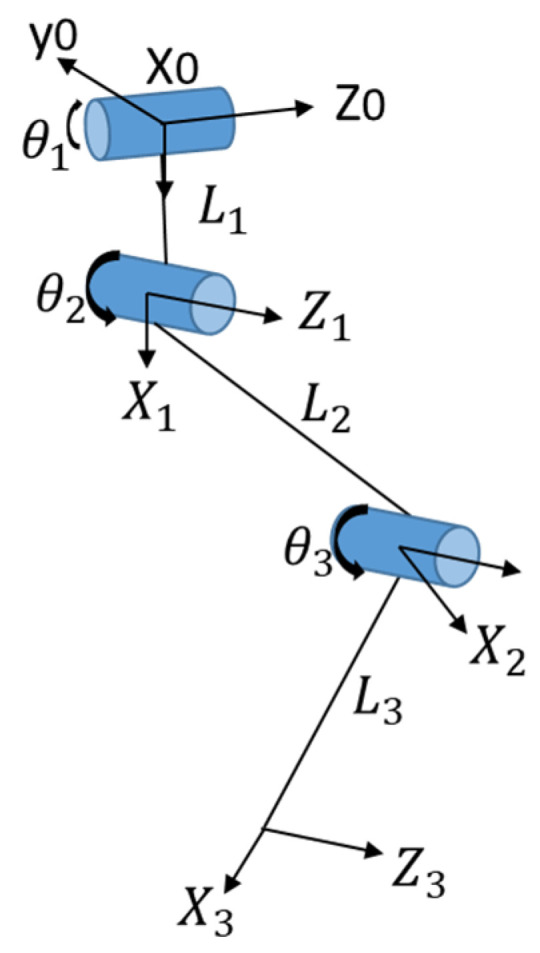
D–H coordinate diagram of leg.

In this paper, the Denavit–Hartenberg (D–H) method is used to analyze the front leg of the robot. A D–H coordinate diagram of the leg is shown in Figure 4.

The homogeneous transformation matrix $T_{3}^{0}$, relating the foot to the hip joint frame, can be represented as follows, where $P_{x}$, $P_{y}$, and $P_{z}$ represent the position of the foot tip in the coordinates of hip joint.

(1) $$T_{3}^{0}=T_{1}^{0}T_{2}^{2}T_{3}^{2}=\left[\begin{array}{cccc} {\cos\theta }_{1}\cos(\theta_{2}+\theta_{3}) & -{\cos\theta }_{1}\sin\left(\theta_{2}+\theta_{3}\right) & {\sin\theta }_{1} & \left(L_{3}\cos\left(\theta_{2}+\theta_{3}\right)+L_{2}{\cos\theta }_{2}+L_{1}\right){\cos\theta }_{1}\\ {\sin\theta }_{1}\cos\left(\theta_{2}+\theta_{3}\right) & -{\sin\theta }_{1}\sin\left(\theta_{2}+\theta_{3}\right) & -{\cos\theta }_{1} & \left(L_{3}\cos\left(\theta_{2}+\theta_{3}\right)+L_{2}{\cos\theta }_{2}+L_{1}\right){\sin\theta }_{1}\\ \sin\left(\theta_{2}+\theta_{3}\right) & \cos\left(\theta_{2}+\theta_{3}\right) & 0 & L_{3}\sin\left(\theta_{2}+\theta_{3}\right)+L_{2}{\sin\theta }_{2}\\ 0 & 0 & 0 & 1 \end{array}\right]$$

(2) $$P_{x}=\left(L_{3}\cos\left(\theta_{2}+\theta_{3}\right)+L_{2}{\cos\theta }_{2}+L_{1}\right){\cos\theta }_{1}$$

(3) $$P_{y}=\left(L_{3}\cos\left(\theta_{2}+\theta_{3}\right)+L_{2}{\cos\theta }_{2}+L_{1}\right){\sin\theta }_{1}$$

(4) $$P_{z}=L_{3}\sin\left(\theta_{2}+\theta_{3}\right)+L_{2}{\sin\theta }_{2}$$

The inverse kinematics can be obtained by Equations (2)–(4):

(5) $$\theta_{1}=\arctan\left(\frac{P_{y}}{P_{x}}\right)$$

(6) $$\theta_{2}=\arccos\frac{L_{3}{\sin\theta }_{3}}{{\left[{\left(P_{x}{\cos\theta }_{1}+P_{y}{\sin\theta }_{1}-L_{1}\right)}^{2}+p_{z}{}^{2}\right]}^{\frac{1}{2}}}-\psi$$

(7) $$\theta_{3}=\arccos\frac{{\left(P_{x}{\cos\theta }_{1}+P_{y}{\sin\theta }_{1}-L_{1}\right)}^{2}+p_{z}{}^{2}-L_{2}{}^{2}-L_{3}{}^{2}}{2L_{2}L_{3}}$$

(8) $$\psi =\arccos\frac{p_{z}}{{\left[{\left(P_{x}{\cos\theta }_{1}+P_{y}{\sin\theta }_{1}-L_{1}\right)}^{2}+p_{z}{}^{2}\right]}^{\frac{1}{2}}}$$

In robotics, the Jacobian matrix is usually used to connect the joint velocity with the Cartesian velocity at the end of the robot. The velocity of the Jacobian matrix J(Θ) can be obtained using Equation (9):

(9) $$V=J\left(\Theta \right)\dot{\Theta }$$

The angular acceleration equation can be obtained by differentiating Equation (9) with time:

(10) $$a_{c}=\dot{J}\left(\Theta \right)\dot{\Theta }+J\ddot{\Theta }$$

In this paper, the Lagrange formulation is used to analyze the dynamics of the robot when the robot moves along a straight line and the hip joint rotates at an angle of $\theta_{1}=0$. 

**Figure 5:**
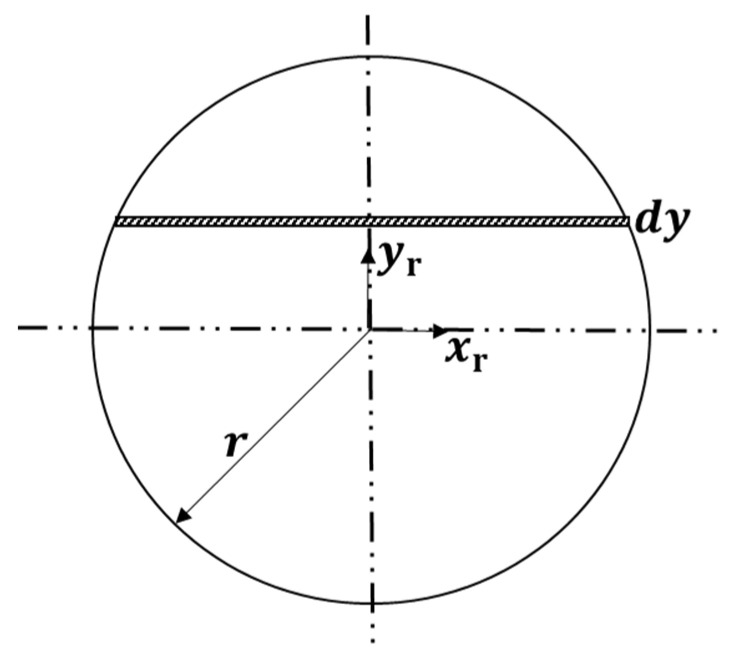
Cross-section diagram of flexible airbag.

**Figure 6:**
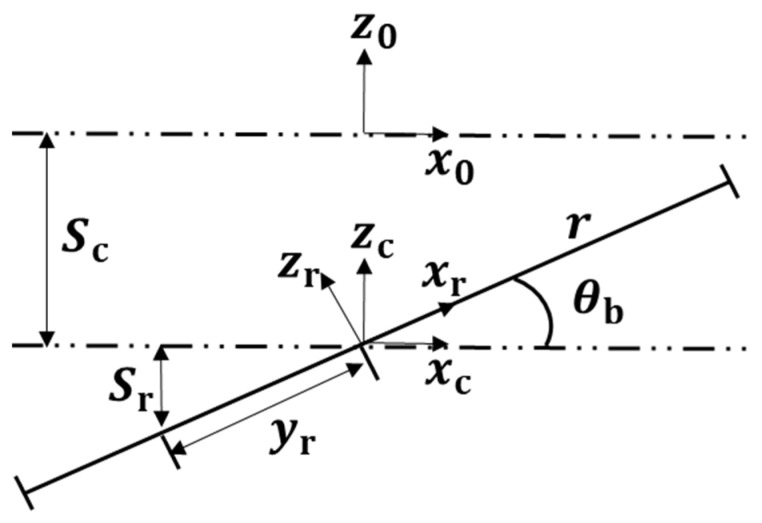
Schematic diagram of potential energy calculation for flexible leg.

As shown in Figure 5, the cross section of the flexible part is equivalent to a circle, and the circular area can be regarded as the area integral of countless rectangles. As shown in Figure 6, because the flexible part is inclined and compressed on the plane, the compression of a rectangle is equal in the same $x_{r}$, which can be regarded as vertical compression. In this way, the inclination can be converted into compression for calculation. 

As shown in Figure 6, the flexible leg can be seen that compressed $S_{c}$ and the recline angle $\theta_{b}$. According to the principle of the mechanics of materials, regardless of other energy loss in the loading process, the tensile and compressive strain energy of the bar is equal to the work performed by the external force w. The compression energy equation of the cuboid is as follows:

(11) $$w=\frac{1}{2}\mathrm{FS}$$

(12) $$E=\frac{F/A}{S/L}$$

Therefore, it can be calculated that:

(13) $$w=\frac{{\mathrm{EAS}}^{2}}{2L}$$

where A is the compression force area, E is the elastic modulus of the material, S is the compression amount, and L is the length of the rod. As shown in Figure 5 and Figure 6, in the case of the same E, L and S, W is only related to A. The results are as follows:

(14) $$W_{r}=\int_{-r}^{r}\frac{E{\left(S_{c}+S_{r}\right)}^{2}}{2L_{3}}\sqrt{r^{2}-y_{r}{}^{2}}{\mathrm{dy}}_{r}$$

(15) $$S_{r}=y_{r}{\sin\theta }_{b}$$

$S_{r}$ is the amount of compression at $y_{r}$ caused by a rotation $\theta_{b}$ of the flexible part. According to Equations (14) and (15), we can obtain:

$$W_{r}=\frac{{\pi \mathrm{Er}}^{4}\sin^{2}\theta_{b}}{16L_{3}}+\frac{{\pi \mathrm{Er}}^{2}S_{c}{}^{2}}{4L_{3}}$$

**Figure 7:**
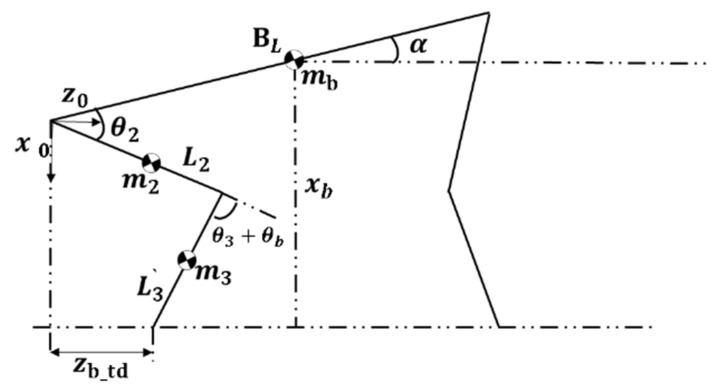
Pose diagram of quadruped robot.

As shown in Figure 7, the center of mass of the legs and body is at its center, according to geometry:

(16) $$L_{3}^{\prime }=\frac{\left(x_{b}-0.5B_{L}\sin\alpha \right)-L_{2}\cos\left(\theta_{2}+\alpha \right)}{\cos\left(\theta_{3}+\theta_{b}-\theta_{2}-\alpha \right)}$$

(17) $$S_{c}=L_{3}^{\prime }-L_{3}$$

(18) $$z_{b\_\mathrm{td}}=L_{2}\sin\left(\theta_{2}+\alpha \right)\cdot \tan\left(\theta_{3}+\theta_{b}-\theta_{2}-\alpha \right)\cdot \left[\left(x_{b}-0.5B_{L}\sin\alpha \right)-L_{2}\cos\left(\theta_{2}+\alpha \right)\right]$$

where $L_{3}^{\prime }$ is the length of the flexible part after deformation, $z_{b\_\mathrm{td}}$ is the displacement of the leg end relative to the hip joint in the z direction, $x_{b}$ is the height of the center of gravity of the robot from the ground, and $B_{L}$ is the body length of the robot, which is solved by Equation (18).

(19) $$\theta_{b}=\arctan\frac{L_{2}\sin\left(\theta_{2}+\alpha \right)-z_{b\_\mathrm{td}}}{\left(x_{b}-\mathrm{bsin}\alpha \right)-L_{2}\cos\left(\theta_{2}+\alpha \right)}+\alpha +\theta_{2}-\theta_{3}$$

After considering the flexible deformation of the leg, the kinematics and inverse kinematics model parameters will be updated with the following inequation:

(20) $$\{\begin{array}{l} L_{3}\leftarrow L_{3}^{\prime }\\ \theta_{3}\leftarrow \theta_{3}+\theta_{b} \end{array}$$

After completing the variable conversion, the final compression energy is only related to the robot’s body inclination, height, and leg end displacement, which can be measured by sensors. The Lagrange function is:

(21) $$L\left(\Theta ,\dot{\Theta }\right)=K\left(\Theta ,\dot{\Theta }\right)-P\left(\Theta \right)$$

The Lagrange dynamic formulation is:

(22) $$\frac{d}{\mathrm{dt}}\frac{\partial L}{\partial \dot{\Theta }}-\frac{\partial L}{\partial \Theta }=\tau$$

(23) $$K=\frac{1}{2}{\mathrm{mv}}^{2}+\frac{1}{2}I^{2}{\dot{\Theta }}^{2}$$

where L—Lagrange multiplier,

τ—Driving torque of the leg,
I—Moment of inertia connecting the rod.

According to Figure 7, it can be concluded that:

(24) $$\{\begin{array}{l} z_{2}=\frac{1}{2}L_{2}\sin\left(\theta_{2}+\alpha \right)\\ x_{2}=\frac{1}{2}L_{2}\cos\left(\theta_{2}+\alpha \right)\\ z_{3}=2x_{2}-\frac{1}{2}L_{3}^{\prime }\sin\left(\theta_{3}+\theta_{b}-\theta_{2}-\alpha \right)\\ x_{3}=L_{3}^{\prime }\cos\left(\theta_{3}+\theta_{b}-\theta_{2}-\alpha \right) \end{array}$$

(25) $$v_{}^{\prime }{}^{2}={\dot{x}}_{}^{2}+{\dot{z}}_{}^{2}$$

In combination with Equations (23)–(25), it can be obtained that:

(26) $$\{\begin{array}{l} K_{2}=\frac{1}{2}(\frac{1}{3}m_{2}L_{2}^{2}+\frac{1}{4}m_{2}r_{2}^{2}){\dot{\theta }}_{2}^{2}+\frac{1}{2}m_{2}({\dot{x}}_{2}^{2}+{\dot{z}}_{2}^{2})\\ K_{3}=\frac{1}{2}(\frac{1}{3}m_{3}L_{3}^{\prime }{}^{2}+\frac{1}{4}m_{3}r_{3}^{2}){\left({\dot{\theta }}_{2}+{\dot{\theta }}_{3}+{\dot{\theta }}_{b}\right)}^{2}+\frac{1}{2}m_{2}({\dot{x}}_{3}^{2}+{\dot{z}}_{3}^{2}) \end{array}$$

The center of mass of rod 2 and rod 3 is at the center of the rod. When $\theta_{2}=\theta_{3}=0$, the system potential energy P is 0, so we can obtain:

(27) $$P_{2,3}=m_{2}g\frac{L_{2}}{2}\left(1-\cos\left(\theta_{2}+\alpha \right)\right)+m_{3}g\left[L_{2}+\frac{L_{3}}{2}-\left(L_{2}\cos\left(\theta_{2}+\alpha \right)+\frac{1}{2}L_{3}^{\prime }{}^{2}\cos\left(\theta_{3}+\theta_{b}-\theta_{2}-\alpha \right)\right)\right]$$

**Figure 8:**
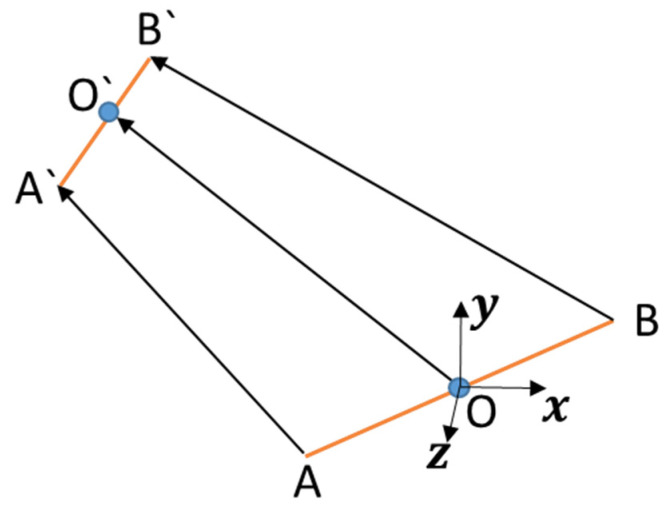
Robot body movement diagram.

As shown in Figure 8, in the diagonal motion state, it is assumed that there is no relative sliding between the foot and the ground during the motion of the robot. A and B are the connection points between the two diagonal legs and the robot body at a certain time, and O is the center of mass of the robot body. At the next moment, the robot moves from AB to A′B′. Where vector $AA^{\prime }={\left({{\mathrm{AA}}^{\prime }}_{x},{{\mathrm{AA}}^{\prime }}_{y},{{\mathrm{AA}}^{\prime }}_{z}\right)}^{T}$, vector $BB^{\prime }={\left(B{B^{\prime }}_{x},BB^{\prime },B{B^{\prime }}_{z}\right)}^{T}$, it is also equal to the mapping of the movement of the end of the leg in the robot body coordinate system, and its value can be calculated by Equations (5)–(7); Vector $AB={\left(B_{L},0,B_{b}\right)}^{T}$, of which $B_{L}$ is the length of the robot body, $B_{b}$ is the width of the robot body, and $m_{b}$ is the body weight.

(28) $$A^{\prime }B^{\prime }=AB-AA^{\prime }+B^{\prime }$$

(29) $$\{\begin{array}{c} \hat{x}=\frac{\sum_{i=y,z}\left(\prod_{j=A^{\prime }B^{\prime },AB}j_{i}\right)}{\prod_{j=A^{\prime }B^{\prime },AB}\left(\sqrt{\sum_{i=y,z}j_{i}{}^{2}}\right)}\\ \hat{y}=\frac{\sum_{i=x,z}\left(\prod_{j=A^{\prime }B^{\prime },AB}j_{i}\right)}{\prod_{j=A^{\prime }B^{\prime },AB}\left(\sqrt{\sum_{i=x,z}j_{i}{}^{2}}\right)}\\ \hat{x}=\frac{\sum_{i=x,y}\left(\prod_{j=A^{\prime }B^{\prime },AB}j_{i}\right)}{\prod_{j=A^{\prime }B^{\prime },AB}\left(\sqrt{\sum_{i=x,y}j_{i}{}^{2}}\right)} \end{array}$$

The space direction offset of the robot’s centroid coordinate, that is, the space angle difference between AB and $A^{\prime }B^{\prime }$, is vector $R_{\mathrm{body}}$;

The space displacement $OO^{\prime }$ of the robot’s centroid coordinate is vector $P_{\mathrm{body}}$;

H=(0,1,0) is the height offset vector of the robot body;

$I_{\mathrm{br}}$ is the moment of inertia matrix of the robot passing through the center of mass;

(30) $$R_{\mathrm{body}}={\left[\hat{x},\hat{y},\hat{z}\right]}^{T}$$

(31) $$P_{\mathrm{body}}=\frac{BB^{\prime }+AB^{\prime }}{2}$$

(32) $$I_{\mathrm{br}}=\left[I_{x},I_{y},I_{z}\right]$$

where

(33) $$\{\begin{array}{l} I_{x}=\frac{m_{b}}{12}\left(B_{L}{}^{2}+B_{b}^{2}\right)\\ I_{y}=\frac{m_{b}}{12}B_{L}{}^{2}\\ I_{z}=\frac{m_{b}}{12}B_{b}^{2} \end{array}$$

(34) $$K_{R}=\frac{1}{2}m_{b}{\dot{P}}_{\mathrm{body}}{}^{T}{\dot{P}}_{\mathrm{body}}+\frac{1}{2}I_{\mathrm{br}}\left({\dot{R}}_{\mathrm{body}}{\dot{R}}_{\mathrm{body}}{}^{T}\right)$$

(35) $$P_{R}=m_{b}gHP_{\mathrm{body}}$$

The total kinetic energy K is equal to:

(36) $$K=K_{2}+K_{3}+K_{R}$$

The total potential energy P equals:

(37) $$P=P_{2,3}+W_{r}+P_{R}$$

According to Equations (21)–(37), the driving torque of the leg τ can be calculated.

### 2.4. Gait Planning and Simulation

**Figure 9:**
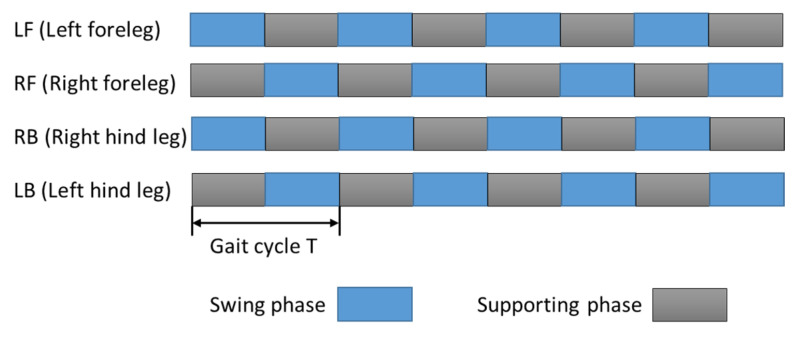
Trot gait phase diagram of quadruped robot.

In this study, we mainly analyze the trot gait. The trot gait is a gait featuring diagonal legs moving simultaneously, also known as “diagonal gait”, which takes into account both stability and speed. It is one of the more common types of gait. A gait phase diagram is shown in Figure 9, and its phase duty cycle of a gait cycle is β =0.5. In the first half of the cycle, when the left front leg and right rear leg swing, the left rear leg and right front leg are supported, and after the second half of the cycle, the left front leg and right rear leg are supported, and the left rear leg and right front leg are in the swing state. The forward motion of the quadruped robot can be realized by cyclic motion.

In the following design of the foot trajectory, as shown in Figure 4, the backward direction is the positive Z direction and the vertical downward direction is the positive X direction. The gait is described by the mathematical model of Equations (29) and (30), where S is the step length and H is the step height.

(38) $$\{\begin{array}{l} z_{t=0}=0\\ z_{t=T/2}=-S\\ z_{t=T}=0 \end{array}$$

(39) $$\{\begin{array}{l} x_{t=0}=0\\ x_{t=T/4}=-H\\ x_{T/2<t<T}=0 \end{array}$$

The phase stability is very important during the phase switching. According to the zero impact principle, the trajectory needs to meet the following constraints in order to achieve zero velocity and acceleration when touching the ground and at the highest position.

(40) $$\{\begin{array}{l} {\dot{z}}_{t=0}=0\\ {\dot{z}}_{t=T/2}=0\\ {\dot{z}}_{t=T}=0 \end{array}$$

(41) $$\{\begin{array}{l} {\ddot{z}}_{t=0}=0\\ {\ddot{z}}_{t=T/2}=0\\ {\ddot{z}}_{t=T}=0 \end{array}$$

(42) $$\{\begin{array}{l} x_{t=0}=0\\ {\dot{x}}_{t=T/4}=0\\ {\dot{x}}_{T/2<t<T}=0 \end{array}$$

(43) $$\{\begin{array}{l} {\ddot{x}}_{t=0}=0\\ {\ddot{x}}_{t=T/4}=0\\ {\ddot{x}}_{T/2<t<T}=0 \end{array}$$

In order to make full use of the constraints of Equations (38)–(43), this paper uses a piecewise quintic polynomial trajectory to solve the foot trajectory equations of the swing phase and support phase. The basic curve is:

(44) $$Z={\mathrm{At}}^{5}+{\mathrm{Bt}}^{4}+{\mathrm{Ct}}^{3}+{\mathrm{Dt}}^{2}+\mathrm{Et}+F$$

When the constraint conditions are brought to Equation (44) according to the phase, the foot trajectory equations corresponding to the swing phase and support phase can be solved as follows: T0 = T/2 = 0.5 s. The swing phase is:

(45) $$\{\begin{array}{l} x=-\frac{6S}{T_{0}{}^{5}}t^{5}+\frac{15S}{T_{0}{}^{4}}t^{4}-\frac{10S}{T_{0}{}^{3}}t^{3},0\leq t\leq T_{0}\\ Z=\{\begin{array}{l} -\frac{192H}{T_{0}{}^{5}}t^{5}+\frac{240H}{T_{0}{}^{4}}t^{4}-\frac{80H}{T_{0}{}^{3}}t^{3},0\leq t\leq \frac{T_{0}}{2}\\ -\frac{192H}{T_{0}{}^{5}}{\left(T_{0}-t\right)}^{5}+\frac{240H}{T_{0}{}^{4}}{\left(T_{0}-t\right)}^{4}-\frac{80H}{T_{0}{}^{3}}{\left(T_{0}-t\right)}^{3},\frac{T_{0}}{2}\leq t\leq T_{0} \end{array} \end{array}$$

The supporting phase is:

(46) $$\{\begin{array}{l} x=-\frac{6S}{T_{0}{}^{5}}{\left(2T_{0}-t\right)}^{5}+\frac{15S}{T_{0}{}^{4}}{\left(2T_{0}-t\right)}^{4}-\frac{10S}{T_{0}{}^{3}}{\left(2T_{0}-t\right)}^{3},T_{0}\leq t\leq 2T_{0}\\ Z=0,T_{0}\leq t\leq 2T_{0} \end{array}$$

**Figure 10:**
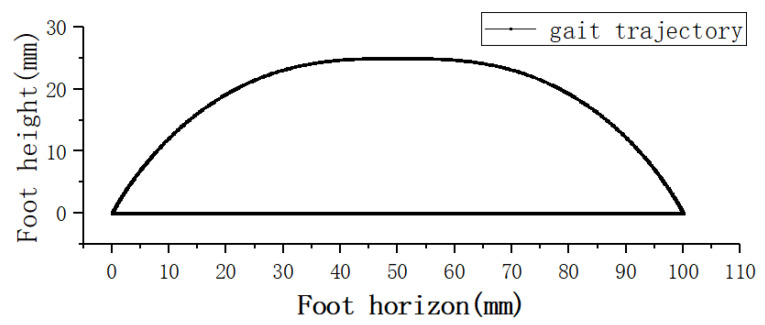
Gait trajectory of robot.

The gait trajectory of the robot is shown in Figure 10. In real control, the relationship between the joint angle and gait trajectory can be obtained by calculating the inverse kinematics [25].

**Figure 11:**
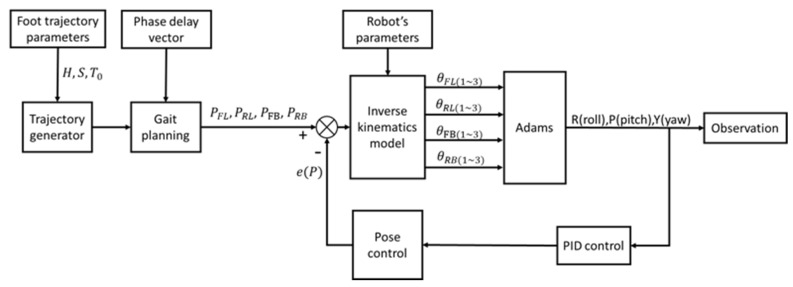
Control flow chart.

The simulation of the quadruped robot is carried out jointly by Adams and MATLAB/Simulink. Adams is the simulation environment, while the controller of the simulation model is in Simulink. The control flow is shown in Figure 11. Two control groups are set in the simulation experiment: a rigid simulation group and a flexible simulation group under the same PID closed-loop control. The flexible part transforms the rigid leg into a flexible leg through the rigid–flexible conversion module in Adams. The interaction time in the Adams interaction module is set to 0.0005 s, the simulation solution of Simulink is set to the variable step, the max step size is 0.0005 s, the simulation time is 12 s, and the gait cycle is $T_{0}=0.8\;s$. The simulation parameters are consistent with the actual structure parameters, step height H = 25 mm and step size S = 100 mm.

**Figure 12:**
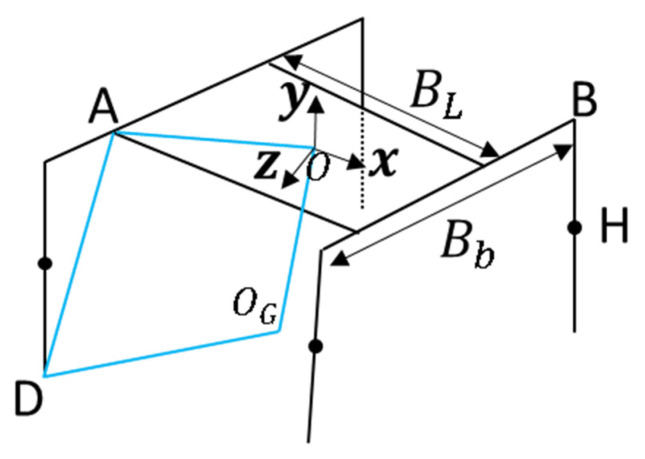
Vector diagram of robot body posture control.

As shown in Figure 12, each leg and body of the robot form a quadrilateral. $OO_{G}$ can be calculated from the rotation matrix $R_{\mathrm{body}}$ and displacement matrix P of the robot. AO can be obtained from the structural parameters of the body. $DO_{G}$ is the vector of D relative to the world coordinate $O_{G}$.

(47) $$AD=AO+OO_{G}-DO_{G}$$

AD represents the position of D relative to A in the coordinate system of point A. Under the post control, in order to ensure the stability of the robot body in the three rotation directions, the position information parameters of vector AD are input into the inverse kinematics model as the error e(P) together with the results of the corresponding leg motion obtained from the gait planning. The corresponding rotation angle of each actuator is obtained for the motion control of the robot.

### 2.5. Detailed System Hardware

**Figure 13:**
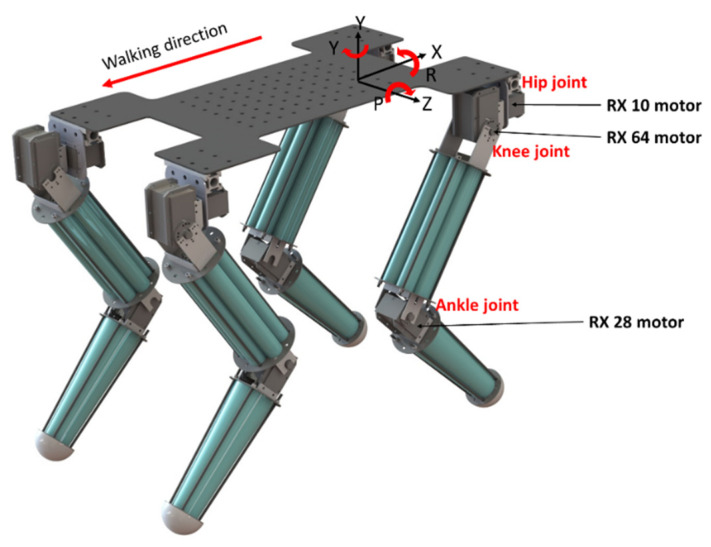
Three-dimensional model of the quadruped robot.

The proposed rigid–flexible coupled quadruped robot model is shown in Figure 13. Other parameters of the quadruped robot are shown in Table 1. One leg is composed of three motors with three degrees of freedom. The inner knee elbow structure is symmetrically distributed in the front and back, which can greatly reduce the sliding between the foot and the ground, thus improving the stability of the robot. Therefore, the robot in this study also adopts this structure. The RX series servo actuator is used as the actuator. The mechanical legs can be controlled by high mechanical strength, high power, high efficiency, and high bandwidth via the direct drive of the motor [23,26]. Additionally, the body of the robot is made of a carbon fiber board with a thickness of 2 mm. Due to its high strength and light weight, the carbon fiber board is the most suitable choice for the robot.

**Figure 14:**
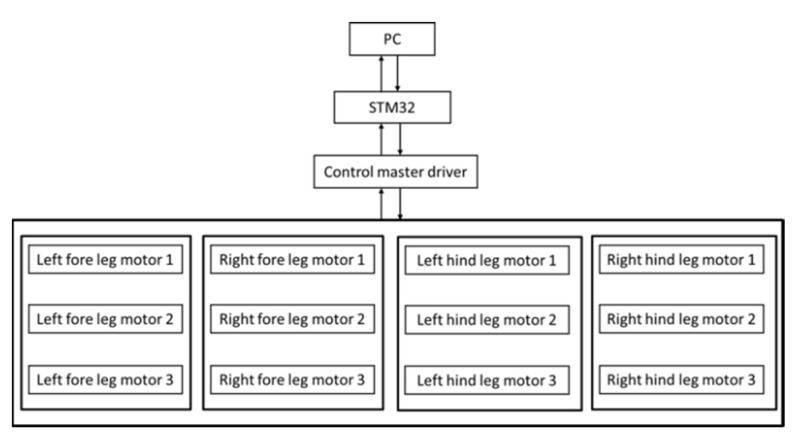
Control chart of the quadruped robot.

STM32f4 MCU with a gyroscope sensor is used for control and can be used as the main board of the quadruped robot, communicate with the computer, and display the parameters of the robot sensor in real time. Additionally, it can be separated from the host computer and controlled directly by the MUC according to the program. The robot drive actuator is equipped with a non-contact absolute encoder. All the same series of the rudders support RS485 serial communication, which is controlled by sending a status package to the ID actuator. The control flow chart is shown in Figure 14. 

When receiving the command, each actuator will return to a status package. The actual rotation angle, torque, voltage, current, and temperature information of the actuator can be obtained from the status package and then returned from the control master driver to the MCU.

## 3. Results

**Figure 15:**
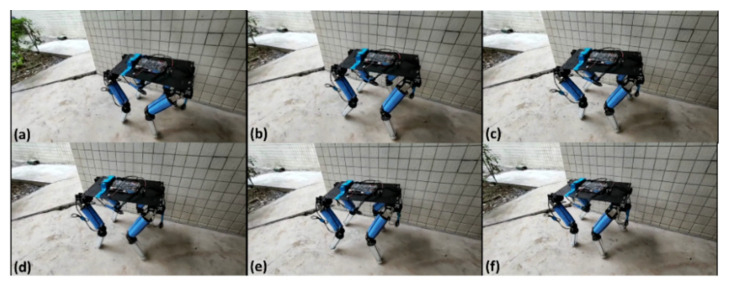
Experiment of real linear motion of robot. (**a**–**f**) represents the motion of the robot.

**Figure 16:**
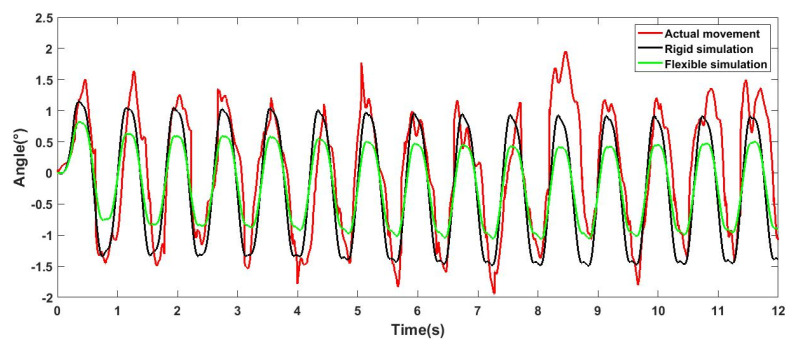
Deviation diagram of robot rolling direction.

**Figure 17:**
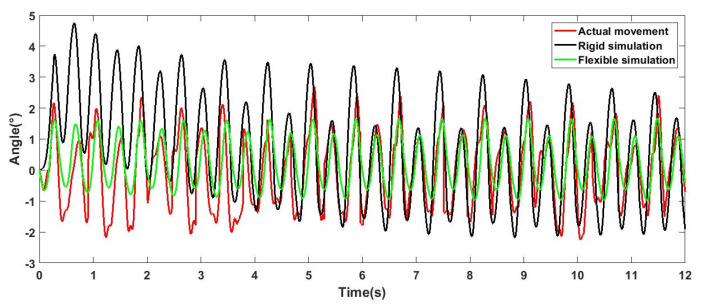
Deviation diagram of robot pitching direction.

**Figure 18:**
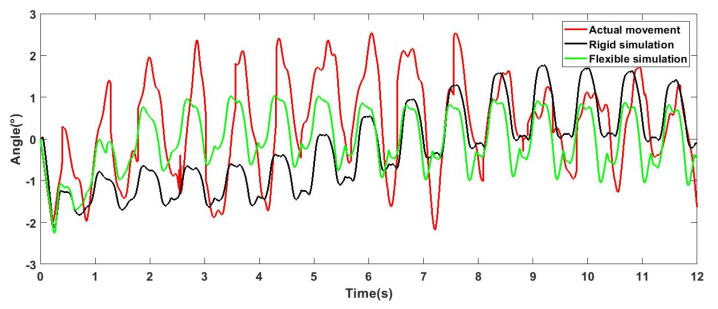
Deviation diagram of robot yawing direction.

The robot carried out a straight-line movement experiment outdoors, as shown in Figure 15 (a to f represents the motion of the robot). On the premise of obtaining relatively stable movement, the movement speed was about 200 mm/s. The experimental results are shown in Figure 16, Figure 17 and Figure 18, which show an offset comparison of the robot in three directions under the rigid simulation, flexible legs simulation, and experimental conditions, respectively.

The results show that the flexible leg has a smaller deflection range than the rigid leg. At 0 s, the robot changes from a standing posture to a trot gait. For the robot, this phase change will bring about the initial error of the body, and the error angle of the flexible leg stabilizes around 0° faster. Moreover, the results show that the flexible part is robust to reduce vibrations and external shock and can also provide better stability via adjusting the stiffness of the flexible leg.

**Figure 19:**
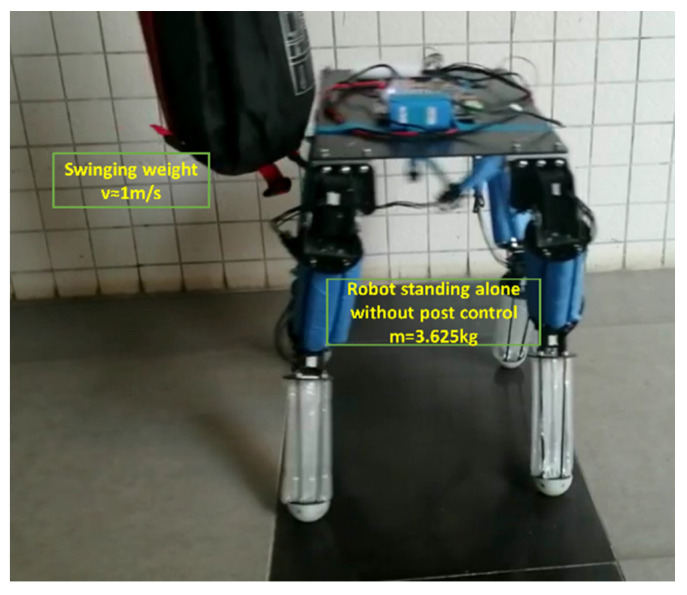
Stability experiment under impact of external heavy objects.

**Figure 20:**
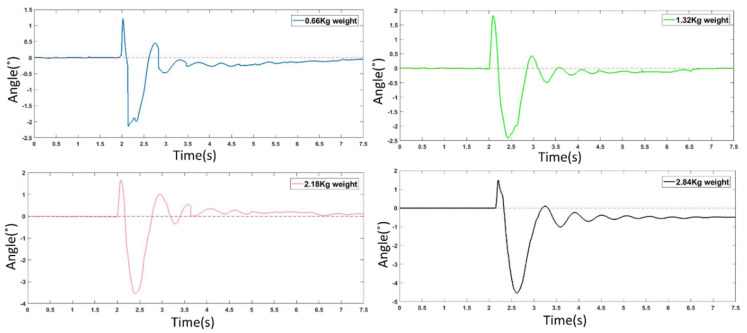
Impact test results.

As shown in Figure 19, during the impact experiment, the robot stands in a normal posture and the side of the robot is impacted with four weights of different masses at a speed of about 1 m/s. Since the quadruped robot does not use any balance algorithm in the experiment, the flexible legs mainly contribute to its stability. The experiments can be seen in video (https://www.bilibili.com/video/BV1QK4y1u7iw/ (accessed on 7 July 2021)) provided in the Supplementary Materials of this research. The experimental results are shown in Figure 20. The abscissa is the time and the ordinate is the rolling angle of the robot. The robot was impacted in the second second. In the impact experiments, the robot had basically completed the buffering of the large impact in 1.5 s. The experiment proves that the structure has good shock resistance and buffering ability. The results are discussed in detail in the next section.

## 4. Discussion

The results in Figure 16, Figure 17 and Figure 18 show that, comparing the simulation results of flexibility and rigidity, it can be seen that the flexible leg makes the robot obtain better stability in the process of motion. The result of actual movement is better than that of rigid simulation, but compared with the simulation results of flexibility, it can be seen that the robot motion error was larger than the simulation result, since there are many challenges in reality, such as the position of the center of gravity, assembly error, and manual error in flexible leg manufacturing (the difference in inflation and the binding of four flexible legs). There are also foot offset errors caused by the flexibility of the slender carbon fiber rods. This experiment is mainly to reflect the advantages of the flexible leg under the same controller. In the control process, the three outputs of the robot roll, pitch, and yaw were adjusted via PID. As the results of the three PID outputs will affect each other, it is difficult to obtain ideal results. In fact, the robot can achieve better walking stability. Experiments proved that the designed flexible leg has better stability, compared with the rigid leg, the amplitude is smaller, and it is faster to stabilize at 0° nearby.

The results in Figure 20 show that in the impact experiments of 0.66 kg, 1.32 kg, and 2.18 kg heavy objects, the robot had basically completed the buffering of the large impact in 1.5 s. In the 2.84 kg impact experiment, there are still some body angle deviations that are not automatically restored. As the impact on the robot is very large, the total weight of the robot body, including all the controllers and the power supply, is 3.625 kg, which is equivalent to being impacted by 80% of the body’s weight. The body had a visible tilt and the legs also deviated from their original position, so there are still some body angle deviations that are not automatically restored but only need a lifting of the flexible leg. When the flexible leg leaves the ground, it can immediately return to its original state. We can also use the complex control algorithm, such as disturbance observers and boundary controllers [27,28], to further optimize the anti-interference and vibration reduction ability of the flexible link. However, the precise control of the flexible body is always a difficulty in the field of control. However, the purpose of replacing the rigid part with the flexible part is to better adapt to the nonlinear uncertain environment. Some environments are difficult to be considered in the control algorithm, so it is worth considering sacrificing part of the control accuracy to improve the flexibility of the robot body structure. Although this will increase the control complexity of the system, it can also have good stability without the complex control algorithm. 

The purpose of this paper is to highlight the advantages of the robot structure itself. This experiment proves the robustness of the rigid–flexible coupled quadruped robot. In the rolling direction, the robot returns to the initial state in a relatively stable form after a short period of oscillation without any balance algorithm.

## 5. Conclusions

This paper introduces a novel rigid–flexible coupled quadruped robot. The flexible leg of the robot is a new three-dimensional flexible structure. The structure is low in cost, lightweight, and features a convenient adjustability of the stiffness and leg length and the robot’s good stability without a complex algorithm. In this paper, the hardware design and software design of the robot are given, the material of the flexible leg is characterized, and the dynamic model of the robot is deduced. After the simulation, walking experiment, and stability experiment, the results show that the designed flexible leg can provide good stability for the robot. We believe that such a robot can provide new ideas for future research on the rigid–flexible coupled quadruped robot. In future work, we intend to use admittance control based on a neural network to achieve the compliant behavior of robotic manipulators in response to external torques from the unknown environment [29,30]. Moreover, we will further increase the number of sensors carried by the robot, and through strengthening the learning to train, further improve the stability of the robot.

## Figures and Tables

**Table 1:** Robot mechanical parameters.

Parameters	
Description	Value
Total mass M	3.625 kg
$\mathrm{Length}\;B_{L}$	0.553 m
$\mathrm{Width}\;B_{b}$	0.2645 m
$m_{b}$	1.021 kg
$I_{x}$	$0.032\;\mathrm{kg}\cdot m^{2}$
$I_{y}$	$0.026\;\mathrm{kg}\cdot m^{2}$
$I_{z}$	$0.006\;\mathrm{kg}\cdot m^{2}$
Hip joint	
$\mathrm{Length}\;L_{1}$	0.029 m
$\mathrm{Mass}\;m_{1}$	0.031 kg
Inertia of hip	$1.5\times 10^{-5}\;\mathrm{kg}\cdot m^{2}$
$\mathrm{Hip}\;\mathrm{joint}\;\theta_{1}$ rotation angle range	−40~60°
Knee joint	
$\mathrm{Length}\;L_{2}$	0.226 m
$\mathrm{Mass}\;m_{2}$	0.376 kg
Inertia of knee	$6.88\times 10^{-3}\;\mathrm{kg}\cdot m^{2}$
Diameter of thigh	0.06 m
$\mathrm{Knee}\;\mathrm{joint}\;\theta_{2}$ rotation angle range	−45~135°
Ankle joint	
$\mathrm{Length}\;L_{3}$	0.218 m
$\mathrm{Mass}\;m_{3}$	0.244 kg
Inertia of ankle	$4.1\times 10^{-3}\;\mathrm{kg}\cdot m^{2}$
Diameter of calf	0.05 m
$\mathrm{Ankle}\;\mathrm{joint}\;\theta_{3}$ rotation angle range	−90~90°

## Data Availability

Not applicable.

## Supplementary Materials

The following videos are available online at https://www.bilibili.com/video/BV1QK4y1u7iw/: Video: A quadruped robot with three-dimensional flexible legs.
